# Inflammasomes in Inflammation-Induced Cancer

**DOI:** 10.3389/fimmu.2017.00271

**Published:** 2017-03-15

**Authors:** Chu Lin, Jun Zhang

**Affiliations:** ^1^Department of Immunology, School of Basic Medical Sciences, Key Laboratory of Medical Immunology, National Health and Family Planning Commission of the People’s Republic of China, Peking University Health Science Center, Beijing, China

**Keywords:** inflammasome, NOD-like receptor, caspase, inflammation, cancer

## Abstract

The inflammasome is an important multiprotein complex that functions during inflammatory immune responses. The activation of inflammasome will lead to the autoactivation of caspase-1 and subsequent cleavage of proIL-1β and proIL-18, which are key sources of inflammatory manifestations. Recently, the roles of inflammasomes in cancers have been extensively explored, especially in inflammation-induced cancers. In different and specific contexts, inflammasomes exhibit distinct and even contrasting effects in cancer development. In some cases, inflammasomes initiate carcinogenesis through the extrinsic pathway and maintain the malignant cancer microenvironment through the intrinsic pathway. On the contrary, inflammasomes also exert anticancer effects by specialized programmed cell death called pyroptosis and immune regulatory functions. The phases and compartments in which inflammasomes are activated strongly influence the final immune effects. We systemically summarize the functions of inflammasomes in inflammation-induced cancers, especially in gastrointestinal and skin cancers. Besides, information about the current therapeutic use of inflammasome-related products and potential future developing directions are also introduced.

## Introduction

Inflammation occurs as a defensive response when a body with vessel system is exposed to invading pathogens as well as physical and chemical hazards. The immune reactions within the body consist of innate and adaptive immune responses (1). Innate immune system has emerged along the evolution of prokaryotes, including tissue barriers, innate immune cells as well as molecules. Unlike adaptive immune system, innate immune system rarely produces immunological memory. Instead, it acts as the first defensive line in a fundamental and unsophisticated way. More importantly, it can sense danger signals and pass them on by intercellular interactions and cytokines (2, 3). Meanwhile, some multiprotein complexes are formed to facilitate the immune responses, for example, apoptosomes and inflammasomes (4, 5).

The recognition of pathogenic components is critical to initiate the host defense system, which requires pattern-recognition receptors (PRRs) (2). Ligands for PRRs are more common and with minor variation, such as lipopolysaccharide (LPS) and heat shock proteins, which are accordingly named pathogen-associated molecular patterns (PAMPs) and damage-associated molecular patterns (DAMPs). Among PRR families, NOD-like receptor (NLR) draws intensive attention for its crucial modulating function to organize a multiprotein complex termed inflammasome (6, 7). The NLR is generally comprised of a central nucleotide-binding and oligomerization domain (NACHT) with dNTPase function, which contributes to conformational changes and autoactivation. In the amino terminus, flanking to NACHT domains, caspase recruitment domain (CARD), pyrin (PYD), baculovirus IAP repeat (BIR), or leucine-rich repeat (LRR) is present and sometimes they coexist (1, 8). The CARD interacts with the initiator caspase-1 and activates the executioners through downstream signaling pathways. PYD belongs to death fold domain superfamily, which is identified as a proapoptotic mediator and function through homotypic interactions. The special motif BIR facilitates the recruitment of adaptor proteins and downstream effectors while LRR, a motif located in the carboxyl-terminus, can sense intracellular PAMPs and DAMPs, similar to its role in toll-like receptors (TLRs).

## Basic Introduction of Inflammasome Family

### Classification

Inflammasome is a multiprotein complex mainly functioning as a platform for the activation of inflammatory caspases which then lead to the maturation of proinflammatory cytokines such as interleukin-1β (IL-1β) and interleukin-18 (IL-18). In addition to proinflammtory cytokines, inflammasome also triggers the secretion of a myriad of leaderless proteins to coordinate cell proliferation and tissue repair (9, 10).

Platform proteins, adaptor proteins, and effector proteins are three key elements assembling inflammasomes. The classification basically depends on the platform proteins, which in turn determine the presence of adaptor proteins and the architecture of the intact multiprotein complex. The platform proteins mainly consist of NLR family as well as HIN-200 protein absent in melanoma 2 (AIM2), whose full name is hematopoietic IFN-inducible nuclear antigens with 200 amino acid repeats (1). NLR family can be divided into three subtypes by polygenetic analysis, including NODs, NLRPs, and IPAFs. Four NLRs proteins, NLRP1, NLRP3, NLRP6, and NLRC4, have been characterized in genetic *in vivo* experiments with immune-deficient mice (11). Interestingly, NLRP3 and NLRP6 do not have CARD domains. Hence, they cannot recruit the initiator caspases directly, which calls for the adaptor protein, apoptosis-associated speck-like protein containing a CARD (ASC), to complete the inflammatory responses (1). ASC is essential for the assembly and the activation of inflammasomes with CARD or PYD through homologous interactions. The NLRC4 inflammasomes with and without ASC are engaged in different pathways and cellular events, respectively, which affirms the regulatory functions of ASC (12). AIM2 is comprised of PYD and HIN-200 domains (9). With PYD, AIM2 can recruit ASC to complete the assembly of inflammasomes.

### Major Mechanisms of Inflammasome Activation

As discussed above, the structures of inflammasomes vary from one to one and therefore the patterns of inflammasome activation differ. Here, we will introduce the major mechanisms of inflammasome activation and enroll the latest discoveries and breakthroughs in this field.

#### NLRP1 Inflammasome

As the first identified inflammasome, NLRP1 recognizes lethal toxin released by *Bacillus anthracis, Toxoplasma gondii*, muramyl dipeptide (MDP) as well as the imbalance of ATP within host cells (13–16). Anthrax lethal toxin is a bipartite macromolecule complex comprised of two proteins. One is protective antigen (PA) and the other is lethal factor (LF) (16). To protect the active component LF from degradation, PA oligomerizes to open up a pore on the cell membrane and transport LF inside. LF is a zinc-dependent metalloprotease cleaving the mitogen-activated protein kinase kinase to hinder MAPK signaling pathway, which eventually results in multisystemic dysfunction (16). The protease property of LF is essential to initiate the activation. In the direct model, LF cleaves the N-terminal region of NLRP1B, releasing NLRP1B from the inactivated state (14, 16). The cleaved NLRP1B recruits caspase-1 through CARD domains. Proximal caspase-1 dimerizes and leads to subsequent maturation of proinflammatory cytokines. In the indirect model, the target of LF is an unknown host factor X which suppresses the function of NLRP1B (14, 16). After the host factor X is processed by LF, the following procedures proceed step by step.

The studies on another important ligand bacterial MDP have been carried out with human NLRP1. It was shown that only when MDP, ATP, and NLRP1 were combined with procaspase-1, cleaved caspase-1 was detectable in the system (15). It suggests that the interaction between bacterial MDP and NLRP1 allow NLRP1 to combine with ATP. NLRP1 mutation study has verified NLRP1 can sense the altered ATP level as metabolic disturbance and can be activated spontaneously (17). Certainly, more experiments are supposed to be conducted to clarify the details in the activation of NLRP1 inflammasome.

#### NLRP3 Inflammasome

NLRP3 inflammasome is one of the best-studied inflammasomes. NLRP3 responds to various activators, a spectrum from microorganisms as well as their derived products, endogenous danger signals to environmental insults. First, NLRP3 can be activated by *Sendai virus, Influenza virus, Adenovirus, Candida albicans, Saccharomyces cerevisiae, Staphylococcus aureus*, and even bacterial pore-forming toxins (18–22). DAMPs like extracellular ATP, hyaluronan, and monosodium urate (MSU) are also ligands for NLRP3 (23, 24). What’s more, amyloid-β can induce the activation of NLRP3 inflammasome in Alzheimer’s patients, which makes NLRP3 inflammasome a hot research target in neurodegenerative diseases (25, 26). Other irritants like silica and asbestos are regarded as environmental insults related to NLRP3 inflammasome activation (24).

Based on the experimental evidence, there are three hypotheses to explain the mechanisms of NLRP3 inflammasome activation. Upon physiological status, NLRP3 is under the auto-suppression with internal interaction between NACHT domains and LRRs. When activators like PAMPs or DAMPs enter the cytoplasm, the auto-suppressive condition is reversed (27). Conformational transformations and recruitments of caspases occur subsequently. An important hypothesis for the activation of NLRP3 inflammasome is the channel and pore-forming model. As is mentioned above, extracellular ATP is a classic activator for NLRP3 inflammasome. It binds to P2X7, an ATP-gated ion channel protein (28), to allow K^+^ efflux and to facilitate the pore formation mediated by a gap junction protein pannexin-1 (29–31). With or without other bacterial pore-forming toxins, the pannexin-1 hemichannel enhances K^+^ efflux and allows extracellular DAMPs and PAMPs to get inside the cell (32). However, for some agonists with big size, like MSU crystals and asbestos, they cannot be transported through the pores. Big size irritants were hard to digest by phagocytosis due to the size and properties. Insufficient clearance of particles results in phagosome disintergration and lysosomal rupture. The damaged lysosome is unable to remain intact and the leakage occurs (33, 34). Cathepsin B is one of the most important proteolytic proteins released by ruptured lysosomes. It is believed NLRP3 is auto-inhibited by protease sensitive proteins under the steady status. When ectopic cathepsin B acts on inhibitory proteins, NLRP3 is reversed from the inhibition. This is called lysosomal rupture model or proteolytic cascade (33). In support of this theory, reduced level of activated NLRP3 was found in human cells that were cultivated with cathepsin B inhibitors. However, it was found cathepsin B inhibitors might function through off-target effects whose real target is NALP1 (35). Thus the accuracy of this theory remains debatable.

If there is a common pathway where different stimuli converge, it is explainable why different pathogens and environmental insults can induce similar immune responses. Fortunately, the reactive oxygen species (ROS) model has been delivered (36). It is believed the production of ROS enhanced by activators can be sensed by NLRP3 directly or indirectly, which leads to its activation (37, 38). This hypothesis is supported by abundant experimental evidence. During the maturation of caspase-1, typical ROS scavengers like *N*-acetyl or P22 (phox) subunit of NADPH oxidase were downregulated (39). Under the resting status, intracytoplasmic thioredoxin-interacting protein (TXNIP) binds to its constitutive inhibitor oxidoreductase thioredoxin (TRX) (40). When DAMPs and PAMPs are transported inside the cell, ROS accumulates, which leads to the disassociation of TXNIP–TRX dimers. Free TXNIP will bind to the corresponding domain in NLRP3, triggering the activation (40). Nevertheless, in another research, the absence of TXNIP did not hinder the activation completely, which implies that other regulatory pathways may be involved. Also, some stimuli related to the production of ROS, for example, ligands for TLRs, sometimes cannot initiate the activation of NLRP3 inflammasome directly (41, 42). It suggests ROS alone may not be sufficient to accomplish the entire activation. Moreover, overproduction of ROS will impair caspase-1 competence by oxidation and glutathionylation. Hence, in order to realize a sound immune response, the production and clearance of ROS should be regulated strictly.

**Figure 1:**
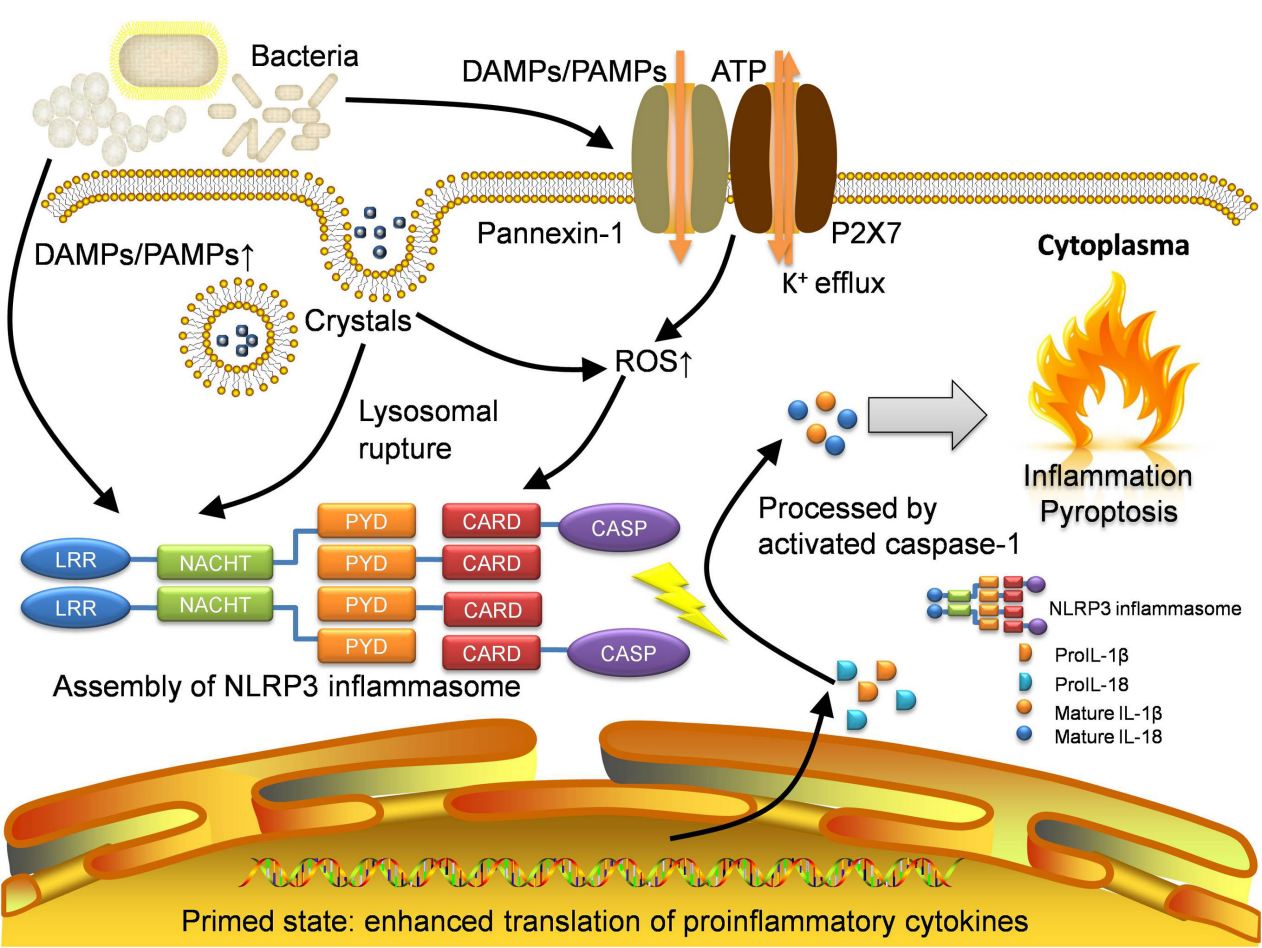
**Activation of NLRP3 inflammasome**. NLRP3 inflammasome can be activated in several approaches. Pathogens especially bacteria will lead to increase of DAMPs and PAMPs, which will be sensed by NLRP3 protein as well as other pattern-recognition receptors like TLRs. The recognition by TLRs will activate NF-κB pathway and allow the host cell to get into the primed state, where the translation of proinflammatory cytokines is enhanced. The open of P2X7, an ATP-gated ion channel, will lead to K^+^ efflux which can also activate NLRP3 inflammasome directly. With the presence of gap junction protein pannexin-1, the influx of DAMPs and PAMPs will also increase. For irritants with large size like MSU crystals, they will be transported through lipid vesicles and merge with lysosomes. The crystals will result in lysosomal rupture and in turn activate NLRP3 inflammasome. Moreover, the production of ROS is increased in the recognition of DAMPs and PAMPs as well as lysosomal rupture. Eventually, intensive oxidative stress also initiates the activation. Assembled NLRP3 inflammasome will cleave raw cytokine materials with activated caspase-1 and release the mature interleukin-1β (IL-1β) and interleukin-18 (IL-18) which cause inflammation and pyroptosis. DAMPs, damage-associated molecular patterns; PAMPs, pathogen-associated molecular patterns; LRR, leucine-rich repeat; NACHT, nucleotide-binding and oligomerization domain; PYD, pyrin; CARD, caspase recruitment domain; CASP, caspase-1; ROS, reactive oxygen species.

Here, we summarize three mainstream mechanisms of NLRP3 inflammasome activation (the channel and pore-forming model, the lysosomal rupture model, and the ROS model) in Figure 1. However, the fact is that different models may function simultaneously during the activation with different extent. Therefore, an integrated model has emerged. Nowadays, with increasing experimental evidence, the integrated theory has been being well acknowledged.

#### NLRC4 Inflammasome

NLRC4 inflammasome has been reported to induce caspase-1 proteolysis and caspase-1-dependent cell death in macrophages (43). So far, many activators for NLRC4 have been identified, such as flagellin from *Salmonella typhimurium, Pseudomonas aeruginosa, Legionella pneumophila*, and *Shigella flexneri* (43–47). In fact, bacterial secretion systems are very important to process and transport the sensitive ligands into cytosol. There are mainly two types of secretion systems in this immune event. One is type III for *S. typhimurium* and *P. aeruginosa* (45, 46) while the other is type IV for *L. pneumophila* (44). Not only flagellin, some needle proteins and inner rod proteins can be also injected inside (48). However, little evidence demonstrated the direct binding between those ligands and NLRC4. For a long time, flagellin is regarded as an essential factor to trigger the activation of NLRC4. Interestingly, some bacteria like *S. flexneri* without flagellin can still be sensed by NLRC4 (47). ASC is dispensable for NLRC4 (49). But in the presence of ASC, the production of IL-1β and IL-18 are enhanced *in vivo*. In macrophages infected with *S. typhimurium*, ASC binds to another ASC and NLRC4 platform protein by homologous interaction (27). Caspase-1, caspase-7, and caspase-8 are recruited into the interspace forming by the NLRC4 inner ring and ASC outside bases (27).

#### AIM2 Inflammasome

Absent in melanoma 2 inflammasome is the first identified non-NLR family inflammasome (50). AIM2 belongs to PYHIN family whose members are featured by PYD and HIN-200 proteins (1). HIN-200 family proteins recognize nucleic acids as activators (51). Unlike NLRP3 inflammasome, AIM2 does not own a CARD, which calls for the assistance of ASC to recruit caspase-1. HIN-200 can sense the cytosolic double-strand DNA from bacterial or viral origins as well as the self-DNA from apoptotic cells (51, 52). Once DNA binds to HIN-200 domain, AIM2 will undergo conformational changes that subsequently contribute to ASC and caspase recruitments.

### Effects of Inflammasome Responses

#### Initiation of Inflammation

Different activated inflammasomes will lead to a common process: the activation of caspase-1. As a result, mature IL-1β and IL-18 will be released. IL-1β is a critical inflammatory cytokine which causes fever of host, activates lymphocytes, and results in local infiltration of neutrophils (53). When IL-1β binds to its receptors, NF-κB and MAPK pathways will be activated, which in turn liberates genes of proinflammatory cytokines from the inhibited state (54). AP-1 and its downstream pathways are also regulated by IL-1β (55). Similar to NF-κB and MAPK, AP-1 also upregulates the secretion of chemokines, adherence molecules, and proinflammatory cytokines. TLR-related pathways regulate the activity of inflammation. The experimental system is habitually primed by TLR ligands such as LPS or proinflammatory cytokines such as TNF before the activation (56). Although, the activation of caspase-1 may also complete without the priming of TNF or LPS, the level of mature IL-1β is pretty low (11, 56, 57). TLR recognition leads to the expression of proinflammatory cytokines like proIL-1β that are induced by LPS or TNF through NF-κB pathway. In one word, TLR pathways prepare the raw ingredients and inflammasome-dependent caspase-1 exerts the cleavage and produces the mature IL-1β.

Another important interleukin cleaved by caspase-1 is proIL-18. Although IL-18 does not cause profound body temperature alteration, it promotes the release of IFN-γ which facilitates the polarization of Th1 subpopulation (58). Analogously, IL-18 also leads to the release of proinflammatory cytokines, chemokines, and synthesis of NO to initiate inflammation (59). What’s more, IL-18 induces the production of Fas ligands for critical apoptosis in immunosurveillance (60).

#### Pyroptosis

Pyroptosis was first reported by Zychlinsky and his colleagues in macrophages infected with *Shigella flexneri* (61). It was initially recognized as apoptosis. But later, it was confirmed as a lytic form of cell death and revised as caspase-1-dependent cell death or another name: pyroptosis (62, 63).

Pyroptosis possesses its own characteristics distinguished from apoptosis. First, pyroptosis is a process of inflammatory cell death accompanied with the release of cytosolic contents while apoptosis rarely leads to inflammation and infiltration of neutrophils (64). In apoptosis, the initiator caspases (capase-2/-8/-9/-10) are activated to cleave the effector caspases (caspase-3/-6/-7) (64). By contrast, upon pyroptosis, it is caspase-1/-4/-5/-11 that is activated to function. In most cases, functions of the initiator and the effector are integrated in identical caspases. Second, the integrity of the cell membrane is disrupted in pyroptosis while the cell membrane remains intact in apoptosis (65). Third, DNA chromatin condensation occurs in both apoptosis and pyroptosis, but nuclear fragmentation is not prominent in pyroptosis and therefore DNA laddering test usually turns out negative (62). However, TUNEL assay is positive in pyroptosis (66). Unlike apoptosis, nucleus stays intact in pyroptosis and the cleavage of ICAD, the inhibitor of caspase-activated DNase, is not present (65, 67).

Activation of caspase-1/-4/-5/-11 will lead to the formation of pores on the cell membrane, which is the final event in pyroptosis. In recent years, Shao with his colleagues endeavored to reveal secrets in the final step of pyroptosis: how the pore is formed and how it damages the cells. They identified gasdermin D (Gsdmd) as the caspase substrate associated with the membrane damage (68). *Gsdmd*-deficient cells cannot undergo pyroptosis even stimulated by LPS or other canonical inflammasome agonists. The experimental results indicated gasdermin-N domains of gasdermin protein such as GSDMD, GSDMA3, and GSDMA can combine with membrane lipids like phosphoinositides and cardiolipin to conduct the pore-forming activity in cells (69). The findings will deepen our understandings of inflammasome-mediated responses.

## Inflammasome, Inflammation, and Cancer

### Convergence of Intrinsic and Extrinsic Pathway

#### Introduction of Intrinsic and Extrinsic Pathway

In a traditional perspective, inflammation is a defensive process against infections and tumors. However, the relationship between inflammation and cancer is more complicated. In fact, inflammation, especially with the participation of inflammasomes, plays a key role in carcinogenesis as well as the promotion of cancer.

Many cancers are linked with chronic inflammation, for instance, gastric cancer from *Helicobacter pylori*-induced gastritis, hepatocarcinoma from *hepatitis B virus* (HBV), or *hepatitis C virus* (HCV) infection and colorectal cancer from ulcerative colitis (UC) (70, 71). The activation of inflammasomes has been documented in many human cancers and the role of inflammasome-related cytokines like IL-1β is also implicated in *in vivo* experiments.

**Figure 2:**
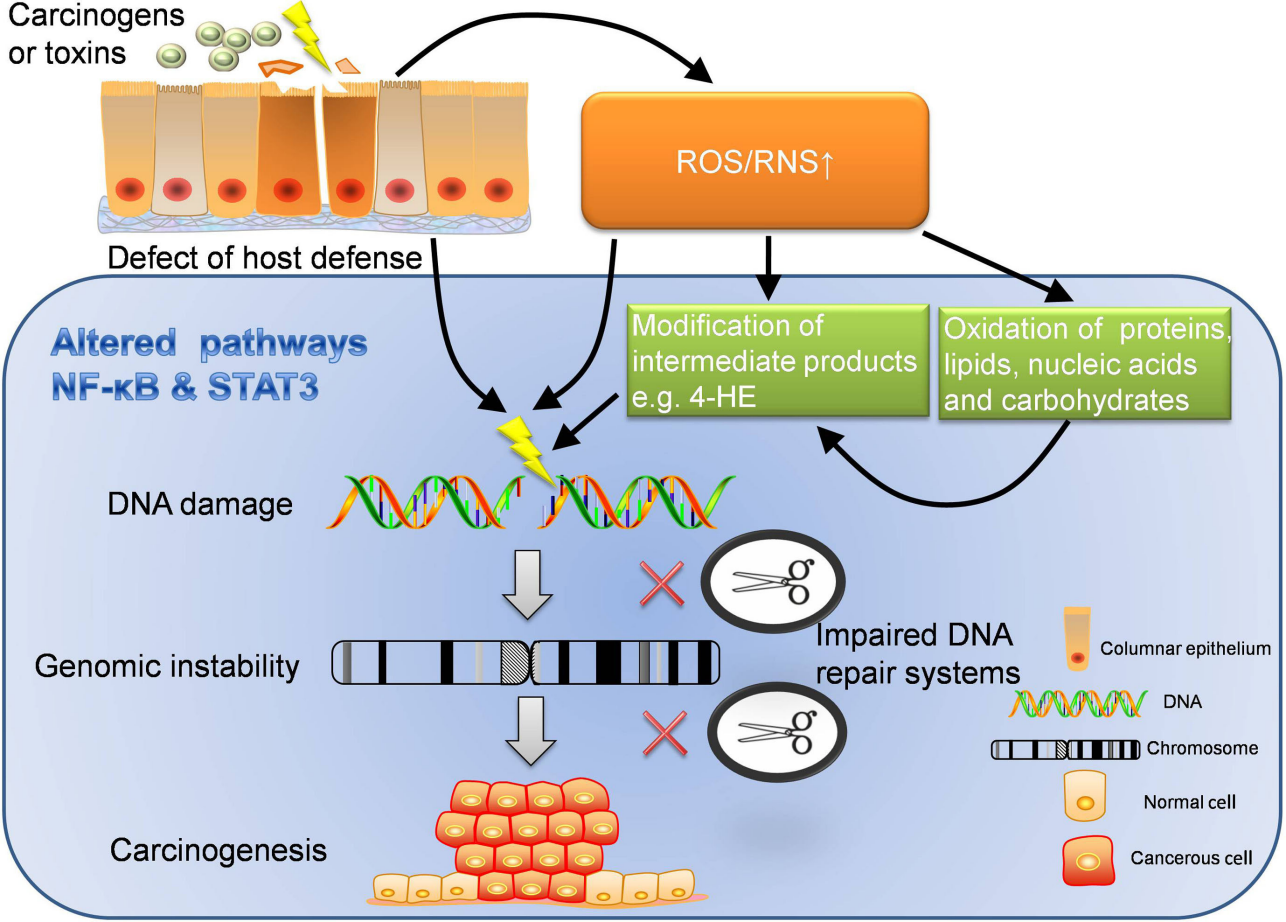
**Extrinsic pathway: inflammation-induced carcinogenesis**. Extrinsic pathway is known as the journey from inflammation to carcinogenesis, which can be interpreted in several dimensions. ROS/RNS can directly damage DNA, which may lead to unlock of oncogenes and inhibition of cancer suppressor genes. In addition, ROS/RNS can oxidize proteins, lipids, nucleic acids, carbohydrates, and worsen the disturbed metabolism. What’s more, intermediate products modified by ROS/RNS also have strong oxidative activity to further damage the DNA. With chronic inflammation, the function of DNA repair system is likely to be impaired. When DNA damage cannot be repaired in time but instead accumulates, genomic instability may occur. If damage persists, carcinogenesis will be the destination. In the perspective of host defense, the defect of immune barriers will increase the risk of contact with carcinogens or other toxins, which also increases the production of ROS/RNS. Furthermore, inflammatory pathways like NF-κB and STAT3 are altered through the whole journey to carcinogenesis. The dysregulated inflammatory pathways are important accomplices behind the scene. ROS, reactive oxygen species; RNS, reactive nitrogen species; 4-HE, 4-hydroxy-2-non-enal.

The connection between inflammation and cancer can be divided into two paradigms: inflammation-induced carcinogenesis, also known as the extrinsic pathway, and cancer-associated inflammation, which is also called the intrinsic pathway (72). Inflammation-induced carcinogenesis is generally initiated by the extrinsic pathway (Figure 2). When cancer is emerged, cancerous cells will activate the intrinsic pathway, realizing the convergence of intrinsic and extrinsic pathway to accelerate the promotion and metastasis of cancer. During the development of cancer, there are four types of inflammation probably involved. They are chronic inflammation caused by infections or autoimmune reactions, inflammation caused by environmental and dietary exposure, therapy-induced inflammation, and cancer-associated inflammation (73). The former two are attributed to the extrinsic pathway. Chronic inflammation, particularly, is a classic predisposing factor for carcinogenesis.

When an inflammasome is activated in face of microbial or environmental insults, it leads to the release of IL-1β and IL-18. Such proinflammatory cytokines continue to attract a myriad of immune cells (e.g., neutrophils, leukocytes, macrophages, and monocytes) to organize a local immune network, which is enhanced by many cytokines, chemokines, and interactions among immune cells. When inflammation is initiated and promoted, the damage to cells and tissues also arises. Now, we will specifically introduce how the extrinsic pathway initiates inflammation-induced carcinogenesis in the following five sections (Figure 2).

#### Carcinogenic Effects of ROS/Reactive Nitrogen Species (RNS)

Under the influence of inflammatory cytokines, the inflamed tissues generate ROS and RNS which are toxic to DNA and contribute to DNA damage (74). When DNA damage occurs where oncogenes or cancer suppressor genes are localized, it will result in the unlock of oncogenes as well as loss-of-function mutations of some cancer suppressor genes (75). Proteins, lipids, and nucleic acids are also likely to be oxidized directly under the high oxidative stress. Moreover, ROS can induce DNA double-strand breaks or DNA cross-links, which leads to replication mistakes. ROS/RNS also facilitates carcinogenesis indirectly by the modification of intermediate metabolic products which mostly are also reactive species. ROS/RNS can modify metabolic products with carbonylation, S-nitrotyrosylation, disulfide formation, or other chemical approaches (72). Such modified products will affect bioactivators like enzymes to alter the cellular or biochemical processes. Lipid peroxidation product 4-hydroxy-2-non-enal is able to cause DNA-adducts and inactivate important cancer suppressor genes like *PTEN* and *STK11* (76, 77).

#### Genomic Instability in Carcinogenesis

Genomic instability in inflamed tissues is usually observed in inflammation-induced cancers. The most common genomic abnormalities are microsatellite instability and telomere changes (78, 79). Genomic instability tends to occur before the inactivation of cancer suppressor genes such as *TP53* (80). So it may be the first genetic event that establishes the basis of carcinogenesis. Microsatellite instability is implicated in many inflammation-cancer transformation models. The proportion of detectable microsatellite instability reaches about 50% in UC patients (81).

Telomere length is a marked distinction between cancerous cells and normal cells. Chronic inflammation may accelerate telomere shortening to induce genomic instability. It was shown that telomere volume expressed a linear correlation with telomere length in gastric carcinoma cells with *H. pylori* infection (82). It suggests carcinogenesis may occur if telomeres are shortened to a critical length. Thus the shortened telomere may be a potential biomarker for carcinogenesis in gastric cancers. Also, shortened telomeres are noticed in colon biopsies from UC compared with nearby tissues (83).

#### Damaged DNA Repair Systems in Carcinogenesis

Chronic inflammation can impair DNA repair systems to enhance carcinogenesis. As is mentioned, inflammation could damage DNA by ROS/RNS directly or through other intermediate oxidative reactive species. Once damage is detected, DNA repair systems will be initiated automatically to correct genetic errors. There are three types of DNA repair systems: base excision repair (BER), nucleotide excision repair (NER), and DNA mismatch repair (MMR). The anticancer effect of BER is demonstrated in dextran sulfate sodium (DSS)-induced colon tumor. AAG, known as alkyladenine DNA glycosylase, is responsible for BER and able to reverse the colon carcinogenesis (84). Furthermore, *Aag*-deficient animals infected with *H. pylori* exhibited severe gastric damage even without the existence of precancerous lesions (84). However, adaptive induction of BER system may lead to microsatellite instability that facilitates carcinogenesis (85, 86). In this regard, the effect of BER on carcinogenesis remains controversial.

High oxidative stress of chronic inflammation also inhibits DNA repair systems. Activated neutrophil-derived myeloperoxidase (MPO) represses the NER pathway in inflamed tissues (87). What’s more, the MPO-processed product hypochlorous acid (HOCl) has similar effects (87). In LPS-induced acute lung injury model, the expression of NER-associated genes like *Xpa* and *Xpf* was decreased (88).

Mismatch repair system is also related to microsatellite instability. There are two key genes in MMR system: *hMSH2* and *hMLH1* (89, 90). The proteins they encode interact with different homologous proteins to conduct repair activity. When inflammatory condition was mimicked by activated neutrophils, colon epithelial cells with different mismatch abnormalities responded differently. Colon epithelial cells expressing hMSH2 displayed G2/M arrest while those do not express hMSH2, p53, or p21 continued the cell cycle (91). *Mlh1* knockout mice displayed higher proportion of colon cancer after the administration of DSS. In colon cancerous tissues, the level of p53 and iNOS was increased and more oxidative DNA damage accumulated (92).

#### Altered Signaling Pathways to Blame: NF-κB and STAT3

When deficient repair systems cannot mend the DNA damage, molecular signaling pathways will be altered as well. Among countless molecular pathways associated with carcinogenesis, NF-κB and STAT3 are most fundamental ones that are constitutively activated in cancerous cells. NF-κB and STAT3 are critical communicating knots to maintain the malignant state. Their target genes are in charge of diverse dimensions of cell life: proliferation, survival, apoptosis, and damage repair (93).

NF-κB consists of a bundle of signal-responsive transcription factors including RelA, c-Rel, RelB, NF-κB1, and NF-κB2 (94). With extracellular stimuli, IκB kinase complex will be phosphorylated and degraded, which releases NF-κB dimer into nucleus to modulate inflammatory and cell survival genes (95, 96). The activation of NF-κB pathway will produce abundant unprocessed cytokines as ingredients for inflammasome-mediated cleavage. The role of inflammasome with NF-κB in cancers has been demonstrated in several experiments. In the murine model of colitis-associated colorectal cancer (CAC), genetic ablation of *Ikkb* results in reduced tumor incidence and size due to the decreased release of cytokines (71). Although NF-κB pathway is a main target in lung cancer, NF-κB inhibitor does not really work out in its treatment. One reasonable explanation is myeloid inhibition of NF-κB strengthens the process of proIL-1β by neutrophils and IL-1β signaling in turn promotes the proliferation of lung epithelial cells to attenuate treatment efficacy (97). If interleukin-1 receptor (IL-1R) antagonist bortezomib is added, tumor formation and growth will be restricted *in vivo*. Another underlying relationship between IL-1β and NF-κB has been also clarified in colon cancer studies. The expression levels of IL-1β, NF-κB (RelA), and miR-181a in colon cancer tissues are higher than those in normal tissues. Experiments have confirmed IL-1β stimulates the expression of miR-181a *via* NF-κB pathway. Increased miR-181a inhibits PTEN to enhance the proliferation of colon cancer cells (98).

Inflammasome effectors may also activate NF-κB target genes directly. Activated inflammasomes lead to the autoactivation of caspase-1 and sequentially arouse caspase-3/-7 as apoptotic or non-apoptotic mediators. It was reported caspase-7 was activated by caspase-1 and translocated to the nucleus after the stimulation of LPS. In this way, caspase-7 cleaved PARP1 which is localized at the promoters for a subset of NF-κB target genes. It suggests inflammasome-dependent caspase activation can regulate the expression of proinflammatory genes through the cleavage of PARP1 (99).

Inflammasome components like ASC can also interact with NF-κB components. Recent evidence implies that ASC has an inflammasome-independent function to activate MAP kinase and NF-κB pathway and eventually enhance the production of non-inflammasome cytokines and chemokines (100).

Apart from induction of proinflammatory cytokines, chemokines (e.g., IL-8), and adhesion molecules (e.g.,VCAM and ICAM), NF-κB also exerts effects on antiapoptotic genes like *Birc2, Birc3, Xiap, Bcl2, Bcl3, Bcl2l1*, cell cycle regulatory protein like cyclin D1 as well as proangiogenic factors like VEGF. Also, NF-κB may downregulate apoptosis-associated genes like *TP53, BAX*, and *BAD* (73).

STAT3 is a member of signal transducer and activator of transcription family which stays inactive without stimuli (101). The activation of STAT3 mainly relies on JAK family members. Once critical tyrosine residues are phosphorylated, STAT3 will undergo dimerization and activate a wide array of target genes (102). Activated STAT3 has been demonstrated in cellular components of cancer microenvironment. Murine models with the depletion of the suppressor of cytokine signaling-3, which is an endogenous inhibitor of JAK–STAT pathway, displayed more colonic crypt formation and increased size of colon tumor after continuous administration of DSS (103). Neoplastic cells and immune cells produce a broad species of cytokines such as IL-6, IL-22, IL-23, and EGF due to the activated STAT3 signaling (104, 105). Besides, the expression of corresponding receptors on membrane surface like EGFR and IL-23R increases as well (106). STAT3 also interacts with other pro-carcinogenesis genes like *Kras, Src*, and *Abl1* whose gene products enhance STAT3 activity in return (107–109). NF-κB and STAT3 function as primary molecular pathways and invite more signaling pathways to join the cross talk, bridging the genetic alterations with phenotype manifestations.

#### Pathological Focus on Host Defensive System

In addition to inflammation-derived damage to the genetic materials, host defense also plays a role in inflammation-induced cancers. This theory was delivered from CAC. There is a huge amount of commensal flora inhabiting in intestines and colons, which is not pathogenic in physiological condition. However, when intestinal epithelium is unable to remain intact from physical, chemical, or microbial insults, the flora will activate NF-κB pathway in macrophages through TLRs. Influenced by proinflammatory factors like prostaglandins, chemokines, and interleukins, the genes tend to mutate. Meanwhile, survival signals are strongly enhanced in transformed epithelial cells. What’s more, incomplete integrity of epithelium increases the intestinal exposure to microbial products which may be carcinogenic and sequentially promotes neoplastic transformation. Hence, favorable host defense is required for anti-carcinogenesis (110).

### Formation of Cancer Microenvironment

**Figure 3:**
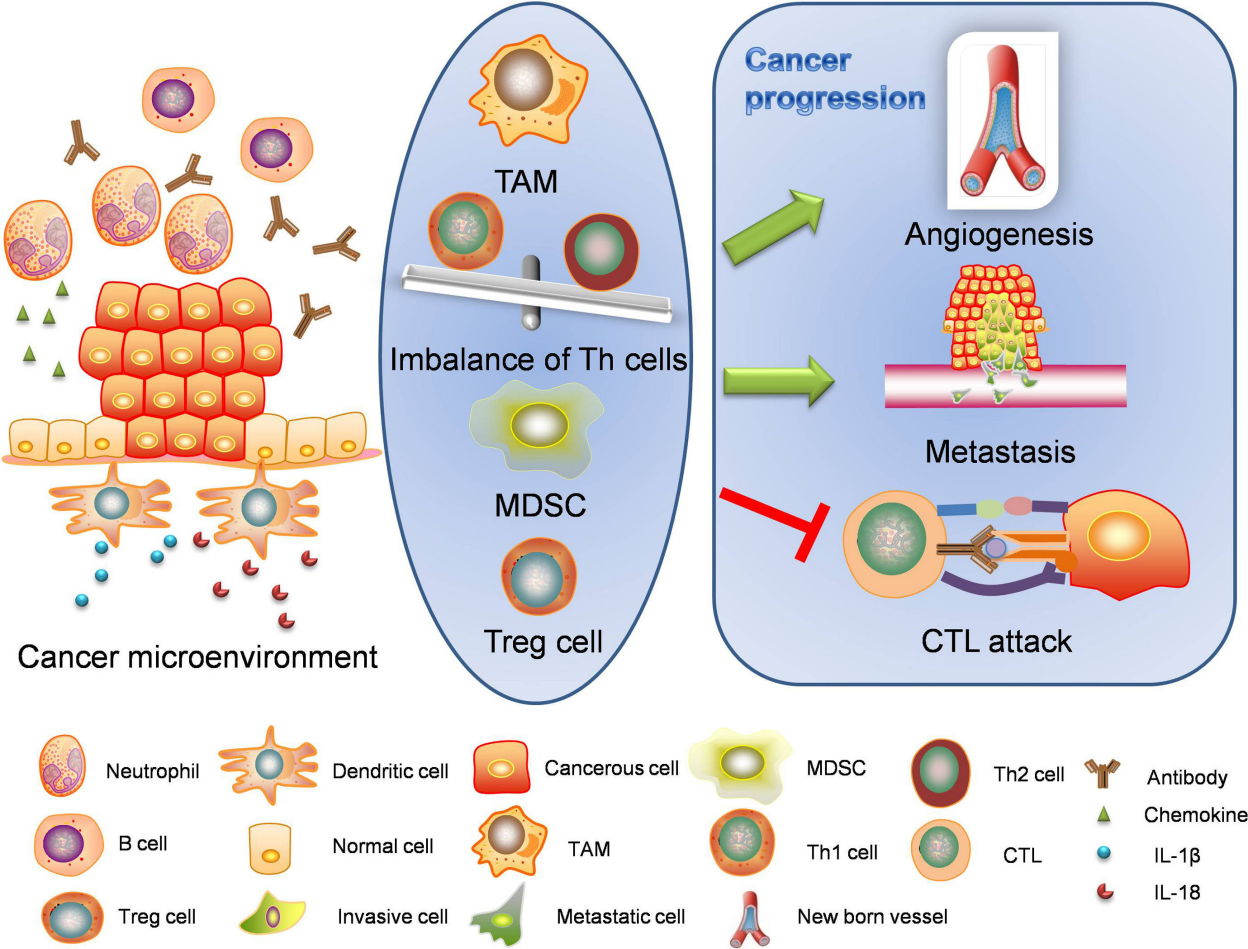
**Intrinsic pathway: cancer-associated inflammation**. Intrinsic pathway is about the formation of cancer microenvironment. The inflammatory cancer microenvironment consists of cellular components and non-cellular components. When transformed or cancerous cells are established, immune responses are also started. Inflammasomes are activated through different mechanisms and release interleukin-1β (IL-1β) and interleukin-18 (IL-18) to initiate inflammation. Cancerous cells and stromal cells can release chemokines and lead to neutrophil infiltration. Neutrophils will in turn secret more proinflammatory cytokines including interleukins and interferons. B cells and antibodies are also observable. Regulatory T cells, TAMs, and MDSCs work together to enhance immunosuppression. The alteration of proinflammatory cytokines will lead to abnormal polarization of T helper cells. The imbalanced T helper cell population and cancer-related immune cells facilitate the cancer progression through angiogenesis, metastasis, and cancerous resistance against immune attacks. TAM, tumor-associated macrophage; MDSC, myeloid-derived suppressor cell; CTL, cytotoxic lymphocyte.

Once premalignant or naïve tumor cells emerge, the tumor itself may initiate cancer-associated inflammation. Cancer-associated inflammation is a classic representative of the intrinsic pathway to organize inflammatory microenvironment for cancers (Figure 3). Cancer microenvironment is critical for epithelial-to-mesenchymal transition, angiogenesis, and metastasis. Inflammatory environment also enhances cancerous resistance against immune attacks.

#### Immune Cells in Cancer Microenvironment

In cancer microenvironment, immune cells exhibit both pro-cancer and anticancer effects. However, especially in chronic inflammation scenario, they show preference in cancer growth and promotion. Tumor-associated macrophages (TAMs) are prime regulators of cancer-associated inflammation (111). Patients with the presence of TAMs are associated with unfavorable prognosis due to their immune suppression functions. Alteration of immune cell polarization has also been reported in inflammation-induced cancers (112). In terms of T helper cells involved, there are two important types of immune responses attracting attention. M1 macrophages usually activate Th1 cells to evoke cellular immunity and shows cytotoxicity while M2 macrophages tend to function with Th2 cells and lead to immunosuppression, angiogenesis, and tissue remodeling activities. Th2 is associated with an array of cytokines like IL-4, IL-5, IL-9, IL-10, IL-13, and TGF-β. In inflammation-induced cancers, M2 type immune response is enhanced, which is known as Th1 versus Th2 switch (111, 112). In circumstance of HBV or HCV infection, a Th2 dominant microenvironment favors persistent inflammation and embraces the transformation from acute hepatitis to chronic liver disease, even hepatocarcinoma (113, 114). However, Th1 immune response claims the predominance in *H. pylori* infection, in which IFN-γ is believed to induce precancerous gastric atrophy, metaplasia, and dysplasia in mice (115).

Another important immune cell involved in suppression of anticancer immunity is myeloid-derived suppressor cell (MDSC). MDSCs are a population of immature myeloid-derived immune cells with potential of differentiation. MDSCs are easy to be detected in patients with high risk of metastasis (116–118). The activity of MDSCs is regulated by inflammation. Evidence showed COX-2 inhibitors were able to reduce the accumulation of MDSCs in Fas-overexpressing tumors (119). Correspondingly, intratumoral inflammation endows MDSCs the resistant property against apoptotic toxicity of Fas/FasL. MDSCs are recruited and regulated by multiple inflammatory mediators such as IL-1β, IL-6, and PGE_2_, which are released by tumor cells and stromal cells in an autocrine or paracrine fashion. In *Il1r*-deficient murine model, the recruitment of MDSCs and tumor progression was repressed and the cancer-associated inflammation in local inflamed tissues was also attenuated. As expected, persistent absence of IL-1R antagonist led to opposite results (120). Like MDSCs, regulatory T cells are also crucial for immunosuppression. They are featured by the high expression levels of multiple immune-checkpoint receptors, such as cytotoxic T-lymphocyte-associated antigen 4 and programmed cell death protein 1 (121). It was shown regulatory T cells can suppress the endogenous interactions between T cells and tumor-antigen-presenting dendritic cells (DCs) in tertiary lymphoid structures to dampen the elimination of tumor cells. In this regard, regulatory T cells can be important therapeutic targets in cancer microenvironment (122).

#### Non-Cellular Immune Mediators in Cancer Microenvironment

Interleukin-1β, as an important inflammasome-processed product, plays a complicated role in cancer microenvironment. Unlike IL-1α, IL-1β is compartmentalized in extracellular environment after cleavage. IL-1β is an important mediator linking innate and adaptive immune responses. IL-1β promotes the polarization of IFN-γ-secreting CD8^+^ T cells and induces generation of IL-17-producing γδT cells (123–125). It was shown dying tumor cells failed to prime IFN-γ-secreting CD8^+^ cells in the absence of functional IL-1 receptors (123). γδT cells are critical immune cell population in innate immune responses. γδT cells are capable of recognizing tumor-associated antigens to conduct anticancer activities (125). However, recent studies are inclined to interpret IL-1β as a pro-cancer factor due to its immunosuppressive and chemoresistant properties. The role of IL-1β in cancer invasion was clarified in B16 murine melanoma models. Apte and his colleagues injected melanoma cells into C57BL/6 wild-type, *Il1a*, and *Il1b* knockout mice, respectively. It turned out carcinogenesis was reduced by 50% in *Il1a* knockout group compared with wild-type groups. Interestingly, there was no tumor formed in *Il1b* knockout group, which highlighted the important role of IL-1 especially IL-1β in carcinogenesis and cancer invasion of B16 melanoma (126). Besides, the polymorphism of IL-1 (IL-1B-31*C/-511*T and IL-1RN*2/2*) is associated with the reduced production of gastric acid and gastratrophia with infection of *H. pylori* (127). Chronic infection with HCV is more likely to proceed into hepatocarcinoma with variable polymorphism of IL-1 and when it comes to pancreatic carcinoma, the prognosis is worse (128, 129).

The activation of IL-1/IL-1R signaling pathway enlists multiple mediators to join the inflammation, such as IL-6, IL-8, TNF-α, and their soluble receptors. IL-6 is an important downstream target gene of NF-κB. Once NF-κB is activated, the expression of IL-6 will be increased. Since IL-6 is a strong inducer of STAT3 pathway, downstream genes of STAT3 will also be activated sequentially. The IL-6–STAT3 signaling axis maintains the intratumoral inflammation and promotes tumor growth. IL-6 released by senescent cells can be sustained and enhanced in autocrine manner while IL-6 promotes the proliferation of surrounding tumor cells in paracrine manner. Bcl-X_L_ and cyclin D1 equip tumor cells with chemoresistance and meanwhile the release of VEGF promotes angiogenesis to guarantee the nutritional supply for tumor cells (72, 130).

In addition to IL-1β, IL-18 is also secreted in inflammatory responses and exerts anti-carcinogenesis effects in colorectal inflammation. The competition between pro- and anticancer cytokines will finally determine the property of cancer microenvironment.

### Anticancer Effects of Inflammasomes

Apart from pro-cancer effects, anticancer effects of inflammasomes are also recorded. First, inflammasome can remove the tumor cells by specialized cell death which is introduced as pyroptosis. As is known, cancerous cells are equipped with the capacity to escape from programmed cell death and get into an immortal phase. Therefore, it is reasonable to speculate pyroptosis is suppressed in carcinogenesis (131). It was reported *Caspase1*-deficient and *Nlrc4*-deficient colon epithelial cells were more resistant to programmed cell death and exhibited increased tumor load compared with wild types (132, 133). It suggests the underlying association between dampened cell-autonomous elimination and local carcinogenesis.

Inflammasome-dependent anticancer effects are more profound when combined with chemotherapies. Immunogenic cell death is highlighted in inflammasome anticancer effects (131). It is a post-tumor effect, which is initiated by tumor-derived molecules but amplified by inflammasomes and other relevant immune cells. When therapeutic approaches damage primary tumor cells, they induce autophagy of tumor cells and lead to the leak of ATP into extracellular space (134, 135). ATP, as an endogenous ligand, can bind to P2Y2 receptors on macrophages, resulting in tumor infiltration (136). Meanwhile it can also bind to P2RX7 receptors on DCs to activate NLRP3 inflammasome (123). Then IL-1β and IL-18 are released and they work together to promote γδT cell-induced secretion of IL-17, which recruits IFN-γ-producing CD8^+^ αβ T cells (131). As a result, IFN-γ finally damages therapy-resistant tumor cells. According to this mechanism, any deficiency within the stimulating axis will attenuate immunogenic cell death. For example, tumor cells which express CD39, a nucleotide-metabolizing enzyme, cannot trigger sufficient anticancer responses. It can be rescued by the administration of extracellular ATPase inhibitors (131). The same situation applies to the deficiency of P2RX7 receptors or use of any specific P2RX7 neutralizing antibody (124, 125). Another study found that tumor-derived autophagosomes, which is also known as defective ribosomal products in blebs (Dribbles), can induce the maturation of DCs and the secretion of proinflammatory cytokines in TLR and NLRP3 inflammasome-dependent manner (137). It was proved to show tumor suppression effects as a therapeutic vaccine in several preclinical cancer models (137). In the model of colorectal cancer metastasis in liver, the activation of NLRP3 inflammasome is able to suppress the metastasis by priming natural killer (NK) cells to enhance immunosurveillance. The maturation of NK cells requires IL-18 which can be provided in inflammasome-dependent responses. Primed NK cells will express Fas ligands on the cellular surface. Once FasL binds to Fas receptor on colon carcinoma cells, apoptosis is started and the cellular components released from apoptotic tumor cells can trigger Kupffer cells again (138).

*Caspase1*-deficient and *Nlrc4*-deficient mice showed reduced apoptosis and enhanced carcinogenesis in CAC models. Interestingly, caspase-1 and NLRC4 mediated direct regulation of cell proliferation instead of inflammatory modification (132). It suggests dominant functions of inflammasomes are different in hematopoietic and non-hematopoietic compartments. In a model of multistage chemically induced squamous cell carcinoma, lack of ASC and caspase-1 led to earlier onset and more papillomas. Disabled inflammasomes resulted in a shift of T cell subsets. For example, CD4^+^CD25^+^Foxp3^+^ T cells were increased. Additionally, the expression level of IL-1β and IL-18 were reduced in tumor lesions (139). In a study of ingenol mebutate treatment for skin cancer, the relapse rate of cancer was profoundly increased in *Myd88*-deficient mice and C57BL/6 mice treated with anakinra compared with control groups. Both deficiencies led to impaired production of IL-1 accompanied with less infiltrated neutrophils. It was implicated IL-1, either IL-1β or IL-α can enhance tumor regression effects of ingenol mebutate through recruiting neutrophils and prolonging their lives (140).

Absent in melanoma 2 is another important component of inflammasomes involved in carcinogenesis, cancer progression, and cancer metastasis. It functions in both inflammasome-dependent and inflammasome-independent manner. Studies showed the expression of AIM2 was downregulated in hepatocrcinoma and the absence of AIM2 was more susceptible to cancer progression (141). Deeper investigations elucidated mTOR–S6K1 pathway is the regulating target of AIM2. Loss of AIM2 leads to overactivation of mTOR–S6K1 pathway and cancerous cells show uncontrolled proliferation and enhanced invasion in progression (141). AIM2 is also associated with lower risk of colorectal cancer. A possible explanation is AIM2 limits Akt phosphorylation through interaction with DNA-dependent protein kinases to regulate the life cycle of epithelial cells (142). Besides, tumor-initiating stem cells with aberrant Wnt pathway replicated rapidly in *Aim2*-deficient models and dysbiotic gut mirobiota has further worsened the situation (143). However, inflammasome-dependent secretion of proinflammatory cytokines was normal. Therefore, AIM2 can also regulate stem cells proliferation against cancer in an inflammasome-independent manner.

Furthermore, the roles of inflammasome are also demonstrated in genetic analysis. IL-1β rs-1143643, NLRP1 rs-11651270, NLRP3 rs-10754558, and IL-18 rs-1834481 are associated with protection against persistent *human papillomavirus* (HPV) infection or HPV-related cervical cancer (144). It further validates anticancer effects of inflammasomes in a genetic perspective.

## Transformation between Inflammation and Cancers: Relevant Diseases

Inflammation-induced cancer is a classic example to study how inflammasomes influence inflammation and cancers. In this part, we revisit previously published experiments and reveal immune roles of inflammasomes in typical inflammation-induced cancers, especially gastrointestinal and skin cancers (Table 1). Furthermore, temporal and spatial differences of immune effects by inflammasomes are also addressed in specific cancers (Table 2).

**Table 1:** **Main roles of inflammasomes in inflammation-induced cancers**.

Inflammation-induced cancers	Inflammasome components or murine models	Outcomes	Possible mechanisms	Reference
Gastric cancer	Interleukin-1β (IL-1β)	Loss of parietal cells, gastric atrophy, metaplasia, carcinogenesis, and cancer growth	(a) Hypoacidity gastric environment with suppressed acid secretion (b) Epigenetic silence of tumor suppressor genes (c) Differentiation of T helper cell immune responses	(149–156)
	Interleukin-18 (IL-18)	Prevention of excessive inflammatory responses	Suppressed Th17 responses	(156)

Colitis and colon cancer	Nlrp3-deficient mouse	(a) Increased susceptibility of dextran sulfate sodium-induced colitis and colorectal carcinogenesis (b) Milder colitis-related symptoms	(a) Reduced secretion of IL-18 and β-defensin (b) Reduced secretion of IL-1β and infiltration of immune cells	(164–166, 178, 181) (167–169)
	NAIP/NLRC4 inflammasome	Protective enteric immune responses and tumor suppression	Increased secretion of IL-18 and maintenance of epithelial integrity	(170)
	Nlrc4-deficient mouse	(a) Increased tumor load unrelated to inflammation (b) Dysbiosis and microbiota translocation	(a) Increased epithelial proliferation and reduced apoptosis (b) Absence of discriminators for gastrointestinal bacteria	(131, 169, 170, 184)
	Nlrp6-deficient mouse	Microbiota difference and increased susceptibility of CAC	(a) Dysregulation of gastrointestinal microbiota (b) Alteration of Notch and Wnt pathways	(168, 182, 183)
	IL-1β	Enhanced carcinogenesis when negative regulation is absent	Not characterized	(184)
	IL-18	Protective effects against colitis while overexpression leads to chronicity	Not characterized	(161, 178)
		Biphasic effects in CAC	Consistent with biphasic effects of IFN-γ	(187–189)

Skin cancer	NLRP1/NLRP3 inflammasome	Sunburn-like inflammation	Activated inflammatory cascade	(198, 199)
	Caspase-1	Removal of cancerous cells and suppressed carcinogenesis	Caspase-1-dependent apoptosis	(196)
	Myeloid cell-specific ASC depletion	Protective effects against carcinogenesis	Not characterized	(201)
	Keratinocyte-specific ASC depletion	Increased susceptibility of carcinogenesis	Regulation of keratinocyte proliferation through p53	

Melanoma	Inhibition of NLRP3 inflammasome	Suppressed migration of melanoma	Reduced secretion of IL-1β and IL-18	(206)
	IL-1β	Increased metastasis potential of melanoma	Silence of differentiation factors in melanoma	(208)
	ASC	Biphasic effects in primary and metastatic melanoma	Consistent with the secretory features of IL-1β in melanoma	(210)

**Table 2:** **Temporal and spatial differences of inflammasome effects**.

	Inflammasome components	Compartments or phases	Effects or activating requirements	Reference
Compartmental differences	NLRP6 inflammasome in CAC	Hematopoietic compartment	More important for host defense despite relatively lower expression compared with epithelium	(182)
	NLRC4 inflammasome in CAC	Non-hematopoietic compartment	Important for anticancer effects	(131)
	ASC in skin cancer	Absence in myeloid cells	Anticancer effects	(201)
		Absence in keratinocytes	Increased susceptibility of skin cancer	

Phasic differences	Interleukin-18 in CAC	Early stage	Enhanced colitis-induced proliferation	(188, 189)
		Later stage	Suppression of proliferation	
	The secretion of interleukin-1β in melanoma	Early stage	IL-1R signal and a costimulator	(203)
		Intermediate stage	Only IL-1R signal	
		Late stage	Autonomous secretion in an positive cycle	
	Silence of ASC in melanoma	Primary melanoma	Reduced cell death and increased cell viability	(210)
		Metastatic melanoma	Anticancer effects	

### *Helicobacter pylori* and Gastric Cancer

*Helicobacter pylori* are Gram-negative bacteria associated with peptic ulcers and chronic gastritis which may progress into neoplasm. It is quite common that *H. pylori* coexist with the human body, mostly colonizing on gastric mucosa. From *H. pylori*-related chronic gastritis to gastric cancer is a classic example of inflammation-cancer transformation initiated by microbial infections (145).

*Helicobacter pylori* are equipped with chemical substances and virulent particles to maintain their special niche on stomach, such as urease enzymes, vacuolating toxin A (Vac A), cytotoxin-associated gene pathogenicity island (cagPAI), and other outer membrane proteins, some of which act as oncoproteins during carcinogenesis directly or indirectly (146). During the development of chronic gastritis, profoundly elevated level of IL-1β is detected and recognized as a critical immunopathological change in gastric carcinogenesis. The alteration of IL-1β is inflammasome dependent. It was confirmed *H. pylori* stimuli can induce caspase-1-mediated cleavage of proIL-1β and proIL-18 with the help of NLR family members such as NLRP3, NLRC4, NLRP6, NLRP7, and NLRP12 (145, 147, 148). K^+^ efflux, phagocytosis, and production of ROS are three typical triggers for the activation of NLRP3 inflammasome. These phenomena were captured in cells under the treatment with live bacteria or *Helicobacter* extracts like p58 unit of Vac A (148). There is another study conducted in murine bone marrow-derived dendritic cells demonstrating a TLR-2-/NOD-2-mediated activation in cagPAI-dependent fashion. It was shown infected *Nod2*^−/−^, *Tlr2*^−/−^, and double deficient murine DCs showed significant reduction of NLRP3 activation (149). Encoded by cagPAI, T4SS is a type IV secretion system for transporting CagA protein into gastric epithelial cells, which provides the second signal for the assembly of inflammasome. CagA can induce the reduction of IκB activation and subsequently lowers the threshold of NF-κB to enhance inflammation (150). On the other hand, the incidence of precancerous dysplasia in DSS-treated transgenic mice is increased, implicating the enhanced carcinogenesis in stomach (150). Therefore, oncoprotein CagA contributes to mutual enhancement of inflammation and carcinogenesis with the infection of *H. pylori*.

Interleukin-1β and IL-18 are two critical effectors related to the prognosis. *H. pylori* evolve to escape from immune attacks, which results in persistent inflammation and persistent high level of IL-1β. IL-1β is able to suppress acid secretion and develop hypoacidity gastric environment. Combined with other cytokines like TNF-α, it leads to loss of parietal cells, subsequent gastric atrophy, metaplasia, and eventually gastric cancer (151, 152). What’s more, IL-1β can reinforce gastric carcinoma growth through ERK1/2 kinase signaling, as the result of the activation of CREB and C/EBPβ (153).

Interleukin-1β is also involved in gastric carcinogenesis by epigenetic modulations. MUC-1 is a cellular surface mucin which is expressed in epithelial cells to maintain the integrity of gastric mucosa. In *Muc1*-deficient mice infected with *H. pylori*, high level of IL-1β will increase the activity of methyltransferase and results in aberrant methylation of trefoil factor-2 (*Tff2*) gene, which further impairs the integrity of mucosa (154, 155). As a result, dysplasia may occur. Apart from *Tff2* and *Fmr1, Hprt* and other tumor suppressor genes are also epigenetically silent in gastric carcinogenesis (156). MUC-1 is a negative regulator in the activation of NLRP3 inflammasome. Ng and his colleagues confirmed the existence of MUC-1 in immune cells and also found MUC-1 can downregulate the TLR/NF-κB pathways and the expression of NLRP3 (157). Therefore, both MUC-1 and NLRP3 can be potential targets for blocking gastric dysplasia and carcinogenesis.

While IL-1β triggers differentiation of *H. pylori*-specific Th1 and Th2 cells, which are responsible for immunopathology, another cytokine IL-18 prevents excessive inflammatory responses to keep the balance between proinflammatory and anti-inflammatory responses. *Il18^−/−^* mice displayed severe immunopathology due to uncontrolled Th17 responses (158). As expected, deficiency of IL-1R showed remissive immunopathology and less preneoplastic lesions with reduced Th1 and Th17 responses, though bacteria colonization is higher (158).

Genetic polymorphism also reflects associations between inflammasomes and inflammation-cancer transformation. For example, −511C>T, −31T>C, and +3954C>T gene types of *Il1b* and *Il1rn* are at higher risk of gastric cancer with *H. pylori* infection (151, 159). Recent gene expression analyses reported that CARD-rs11672725, NLRP-rs10754558, NLRP3-rs4612666, NLRP12-rs199475867, and NLRX1-rs10790286 are significantly associated with gastric cancers (160), which again emphasize the importance of NLR signaling pathway in gastric carcinogenesis.

### Colitis and Colon Cancer

Gastrointestinal inflammatory diseases are quite variable and complicated for clinical physicians, among which inflammatory bowel disease (IBD) has close associations with inflammasomes. IBD consists of ulcerative colitis (UC) and Crohn’s disease (CD) (161). The mechanism of IBD is still a mystery. Clinicians prefer to identify IBD as a result of dysregulation of innate and adaptive immune responses, predisposed by dysbacteriosis in guts and other environmental conditions (161). Genetic susceptibility of the host also contributes to its pathogenesis. Both UC and CD can cause abdominal pain, bloody diarrhea, and weight loss and are present as chronically persistent inflammation. UC and CD are mainly distinguished by clinical, endoscopic, and histological criteria. Inflammation is restricted within the mucosal layer in UC especially in rectum and extends continuously to other segments of colons (162). Unlike UC, inflammation is transmural and presents as skip lesion which is discontinued at any part of colons in CD, typically in distal ileum (163). Since inflammatory regions are different, the formation of crypt abscess is common in UC patients while formation of granulomas, fissures, and fistulas is more observable in CD (162, 163).

Recent studies showed inflammasomes and their components play a regulatory role in IBD. Independent research groups confirmed that *Nlrp3*-deficient mice were more susceptible to DSS-induced colitis and relevant symptoms like weigh loss were more severe (164, 165). It was observed that *Nlrp3*-deficient mice showed delayed epithelial renewal and impaired epithelial repair. It may explain the worse clinical presentations in such models (164). NLRP3-mediated IL-18 production was also reduced, which disturbs the intestinal homeostasis (166). However, the protective role of NLRP3 was questioned by other opposite research results. It was reported that *Nlrp3*-null mice exhibited milder colitis-related symptoms after treatment with DSS, probably due to the reduced secretion of IL-1β (167). This phenomenon was confirmed by another research group (168). Such contradiction may be explained by different microbiota in different strain of mice as baseline bias (169).

Apart from NLRP3, NAIP/NLRC4 is also crucial in maintenance of the integrity of intestinal epithelium (170). NAIP/NLRC4 can respond to Gram-negative bacteria such as *Salmonella* Typhimurium and *Citrobacter rodentium* as well as bacterial components like type III secretion system (TTSS) and flagella (171–173). The exposure to acute carcinogens can also be sensed. Intestinal epithelial cells will activate NAIP–NLRC4–caspase-1 axis which increases the secretion of IL-18 to activate protective gut immune responses (170).

A little different from inflammasomes mentioned above, NLRP6 functions as a regulator of gastrointestinal microbiota. *Nlrp6*-deficient mice harbored quite different gut microbiota from wild-type mice, characterized by an increase of *Anaerobic taxa, Prevotellaceae*, and TM7 but the reduction of *Lactobacillus* (168). Interestingly, the colitogenic microbiota in *Nlrp6*-deficient can be transferred to wild-type mice by cohousing (168). However, this finding is restricted to *Nlrp6*-deficient mice, which does not apply to other inflammasomes such as NLRC4 or AIM2 (169).

Increased IL-1β was noticed in the hematopoietic cells in lamina propia as well as epithelium in IBD patients, which is associated with the severity of the disease and prognosis (174, 175). Treatment with IL-1 blocking agents showed rescuing effects (176, 177). IL-18 shows protective effects in DSS-induced colitis models. It was found that administration of recombinant IL-18 could rescue *Caspase1*-deficient B6 mice from DSS-induced epithelial injury, although the secretion of IL-1β and IL-18 were both impaired in this model (178). However, overexpression of IL-18 may allow colitis to enter a chronic phase (161). In active CD patients, the secretion of IL-18 is reduced in epithelium but increased in macrophages. This shift of the secretion in different cells may influence the progression of the disease (161).

Colitis-associated colorectal cancer (CAC) is the most severe complication of IBD (179). Researchers redesigned an azoxymethane (AOM)/DSS model to study CAC. In this model, experiment animals are injected with carcinogen AOM plus two to three rounds of DSS treatments (180). Using this model, the role of inflammasomes in CAC is getting characterized. It was shown *Nlrp3*/*Caspase1*-deficient mice exhibited increased susceptibility of colorectal carcinogenesis when chronic inflammation was enhanced in AOM/DSS model (165, 178). What’s more, colitis-associated adenomatous polyps were observed in *Caspase1*-deficient mice while absent in *Nlrp3*-deficient mice (133). And the extent of protective effects relatively relied on the concentration of DSS used. Another protective mechanism was indirectly proved by impaired β-defensin production in *Nlrp3*-deficient models, which will in turn change the composition of microbiota in guts (181). However, some groups got contradictory results that *Nlrp3*-deficient mice treated with DSS exhibited attenuated colitis and reduced infiltration of immune cells (169). The role of NLRP3 in CAC still needs more elucidations.

Later studies demonstrated the role of NLRC4 and NLRP6 in CAC. Although the expression of NLRP6 is higher in epithelium and lower in hematopoietic cells, the NLRP6 activated in hematopoietic compartment is more important for host defense against the CAC development (182). In *Nlrp6*-deficient murine models, alteration in Notch and Wnt pathways was noticed (183). Although *Nlrp6*-deficient mice grew normally, they developed crypt hyperplasia spontaneously, accompanied with altered crypt-to-villus ratio in distal ileum and enlargement of Peyer’s patches (161). All these pathological presentations increased the susceptibility of CAC. The deficiency of *Nlrp6* will lead to the alteration of microbiota composition in guts as well (170). With the reduced secretion of IL-18, the progression of CAC will be accelerated. It was confirmed *Nlrp6*-deficient mice with AOM/DSS treatment were more susceptible to colorectal carcinogenesis than the wild types (168, 182). The roles of NLRC4 in CAC still remain controversial. Although NLRC4 was implicated to have effects of tumor suppression, another two groups argued that NLRC4 has no roles or even a negative role in anticancer immune responses (133, 165). Moreover, *Nlrc4*-deficient mice of AOM/DSS models exhibited increased tumor load but unrelated to inflammation. It was associated with the increased epithelial proliferation and reduced apoptosis of tumor cells (133). Unlike NLRP6, NLRC4 is more important for anticancer responses in non-hemapoietic compartments (133). NLRC4 is also a discriminator of commensal and pathogenic bacteria in guts, which requires macrophage responses. Therefore, deficiency of *Nlrc4* can result in dysbiosis and microbiota translocation as well (169, 170, 184).

Colorectal carcinogenesis is also associated with dysregulation of inflammasome-related cytokines. In *Nlrp3/Nlrp6*-deficient murine models of AOM/DSS, markedly decreased production of IL-18 were observed (164, 165, 182). In accordance with this result, increased number of tumors was observed in *Il18*/*Il18r*-deficient mice (185). Deficiency of any downstream mediator in IL-18 pathway like MYD88 is more susceptible to intestinal hyperproliferation and carcinogenesis (186). Additionally, administration of recombinant IL-18 can induce remission in colitis-associated injuries and suppress the progression of colorectal tumor in *Nlrp3*/*Caspase1*-deficient mice (166). Reduction of IL-18 secretion is always accompanied with an increase of other proinflammatory cytokines, chemokines, and enzymes such as IL-6, TNF-α, MIP1, MIP2, and MMPs (187). The cytokines like IL-6 and TNF-α may activate pro-tumor pathways like STAT3 to promote cellular proliferation and thereby initiate carcinogenesis (187). Other factors like ROS, RNS, and COX-2 also contributes to the formation and maintenance of cancer microenvironment (187). The biphasic roles of IL-18 in CAC are reported. In detail, IL-18 can enhance colitis-induced proliferation at the early stage but suppress the proliferation at late stage (188). This property is consistent with the biphasic effect of IFN-γ on DSS-associated colitis (189). As an inducer of IFN-γ, IL-18 increases the expression of IFN-γ and then IFN-γ will activate several intrinsic cellular pathways such as JAK–STAT (187). Phosphorylation of JAK1 and JAK2 will subsequently promote the phosphorylation and nuclear translocation of STAT1. In nucleus, STAT1 binds to IFN-γ responsive elements, which are genes in charge of proliferation, differentiation, and cellular death. Therefore, IL-18–IFN-γ–STAT1 axis activation is characterized in carcinogenesis within the colon (166). It was supported by the observation that administration of IFN-γ or IL-18 can rescue reduced phosphorylated STAT1 in *Capapse1*-deficient mice of AOM/DSS models (166).

A recent study revealed that keratin 8, an important filament protein in epithelial cells protected intestinal homeostasis against cellular stress. It was shown the activation of procaspase-1 was increased in *Krt8*-null mice, which leads to the elevated level of IL-18. IL-18 can inhibit the generation of IL-22BP. Therefore, IL-22 is increased in cells, which results in activation of STAT3 cascade (190). The role of AIM2 was also observed in IL-18–IL-22–STAT3 axis. In a steady state, AIM2 inflammasome activation leads to the secretion of IL-18 and activate STAT3 in a similar way. Both IL-18 and STAT3 pathway can positively regulate the expression of antimicrobial peptides Reg3β and Reg3γ to prevent dysbiosis. Therefore, the absence of *Aim2* will result in the dampened production of IL-18 and the decreased expression of Reg3β and Reg3γ (191). However in *Aim2*-deficient mice with DSS-induced colitis, IL-18 is enhanced through other mechanisms, which subsequently lead to sustained activation of STAT3 and Akt pathways. The overexpression of Reg3β and Reg3γ will in turn maintain the activated state of STAT3 and Akt, contributing to dysregulated crypt formation and increased susceptibility of CAC (191). IL-18-enhanced release of proinflammatory cytokines is able to maintain local inflammation and inhibits the translocation of microbiota but activates resident myleiod cells as well as epithelial cells. Proinflammatory cytokines released by such cells tend to be tumorigenic.

Unlike IL-18, IL-1β is not quite relevant to CAC. *Il1r*-deficient mice showed similar number of adenomatous polyps compared with wild-type littermates (184). However, excessive IL-1β also promotes the formation of adenomatus polyp and colorectal carcinogenesis with AOM/DSS when negative regulation is deficient in IL-1 signaling (184). NLRP12 and PYHIN family inflammasomes also show enteric immune regulatory effects in intestinal diseases (161, 192). Non-canonical caspases such as caspase-4 and caspase-11 are also involved in direct recognition of pathogens and the maintenance of gastrointestinal homeostasis (170). The unique NLR family member NLRX1, which does not assemble into an inflammasome, has been recently proven to function as an intrinsic tumor suppressor in intestinal epithelial cells (193).

### Sunburn and Skin Cancer

Skin constitutes a critical external barrier against microbial and non-microbial insults. The outer part of skin, known as epidermis, protects human body by natural stratification. Ultraviolet radiation B (UVB) irradiation is the most common cause for sterile skin lesions (194). Long duration or high volume exposure of UVB will result in inflammatory cutaneous responses, which are likely to progress into skin cancer. Repeated UVB irradiation may alter the characteristics of basal cells and disturb the balance between proliferation, terminal differentiation, and apoptosis within suprabasal layers. UVB irradiation can lead to DNA double-strand breaks and impair DNA repair systems (194, 195). When DNA mutations accumulate over the threshold, cutaneous carcinogenesis is started.

Ultraviolet radiation B irradiation-induced inflammation, or called sunburn-like inflammation, was featured with the increased expression of caspase-1 in mice (196). Also, proIL-1 and proIL-18 have been detected. It revealed the involvement of inflammasomes (197). Later experiments verified the activation of inflammasomes in skin-derived keratinocytes. Actually, in human keratinocytes, proIL-1α/-1β and IL-1Ra are constitutively expressed (198). However, mature proinflammatory cytokines will not be released without stimuli. Small interfering RNA experiments showed that NLRP3 inflammasome is the main mediator responsible for the UVB irradiation-induced secretion of IL-1β, although NLRP1 takes a part as well (198). Strangely, the common triggers of NLRP3 inflammasome such as K^+^efflux and extracellular ATP fail to induce the secretion of IL-1β in keratinocytes (198, 199). Instead, release of intracellular stored Ca^2+^ arouses the activation. The activation of inflammasomes promotes the inflammatory cascade, which is responsible for the sunburn inflammation. Also, cutaneous coexposure of two strong carcinogens: arsenic and UVB irradiation led to more severe epidermal hyperplasia and DNA damage in mice (200). With the analysis of protein expression and cytokine profiling, it confirmed the activation of inflammasomes and the increased expression of proinflammatory cytokines especially IL-1β in coexposure murine models (200). Inhibition of IL-1β signaling pathway can relieve the symptoms and protect cells from carcinogenesis.

As an important component in cutaneous inflammatory responses, caspase-1 also functions independently of inflammasomes. It was noticed that UVB irradiation induced keratinocyte apoptosis was profoundly later than the activation of inflammasomes (196). What’s more, the antiapoptotic molecule Bap31 has been identified as a putative substrate for caspase-1 through proteomic techniques (196). Caspase-1-dependent apoptosis of damaged keratinocytes may be an important way to remove cancerous cells and block the carcinogenesis.

The role of adaptor ASC has been also demonstrated. *Asc*-deficient mice especially with the absence in myeloid cells exhibited protective effects in tumor development, similar to the phenotype in *Nlrp3*/*Caspase1*-deficient mice (201). However, lack of *Asc* in keratinocytes was more susceptible to skin cancer comparing with control groups (201). It highlights the functional difference of ASC in different cellular compartments. ASC is regarded as a tumor suppressor in keratinocytes as its gene is downregulated in cancerous tissues like squamous skin carcinoma while its expression is normal in psoriatic lesions (202). Recently, the mechanism of ASC’s anticancer effects was demonstrated in HaCaT cells. When HaCaT cells were treated with UVB irradiation, the interaction between ASC and p53 was discovered in UVB dose-dependent manner, which led to phosphorylation of p53 and activation of downstream genes (201). It provides a possible explanation for regulatory effects of ASC on keratinocyte proliferation, which makes ASC a potential therapeutic target and carcinogenesis monitoring biomarker.

### Melanoma

Melanoma is another aggressive form of skin cancer. Relationship between inflammation and melanoma has been highlighted in recent researches, especially when the roles of inflammasomes are being explored.

Two important ATP-dependent iron channels, P2X7 and PANX1 are involved in melanomagenesis and tumor progression (203). P2X7 as well as PANX1 is known as typical activators to NLRP3 inflammasome (203). Furthermore, P2X7 also plays a role in stem cell growth and influences cell fate in different ways (204, 205). Opening of P2X7/PANX1 activates the assembly of NLRP3 inflammasome and results in the release of proinflammatory mediators. Inhibition of NLRP3 inflammasome by thymoquinone resulted in reduced secretion of IL-1β and IL-18 with the suppressed the migration of melanoma (206). The level of IL-1β is correlated with metastasis potential of melanoma (207). IL-1β treatment on melanoma cells led to a significant reduction in mRNA expression of microphthalmia-associated transcription factor (MITF-M) (208). The silence of MITF-M would result in dysregulation of gp100 and tyrosinase, which are both important differentiation factors in melanoma (208). As a result, melanoma expressed less anti-melanoma antigens and became “invisible” in immunosurveillance.

Moreover, the secretion of IL-1β is also phase-dependent in melanoma (203). In the early stage, two elements are needed for activation: IL-1R signal and a costimulator, for example, MDP. In the intermediate stage, IL-1R signal is enough to trigger the activation while in the late stage the synthesis and secretion of IL-1β requires no external signals but instead continues in autoactive manner. Autonomous release of IL-1β will in turn enhance the secretion of IL-1β itself, resulting in a positive loop. Hence, inflammasome–caspase-1–IL-1β axis can be targeted for development of anticancer drugs. For example, epigallocatechi-3-gallate from green tea can suppress melanoma growth by inhibiting inflammasome components and the secretion of IL-1β, which can be abolished by silencing the expression of NLRP1 (209). Unlike IL-1β, IL-18 is pretty low in melanoma cells, even undetectable. The role of IL-18 in melanoma progression awaits discoveries (209).

Inactivation of ASC by hypermethylation has been observed in many malignancies like lung, prostate, and breast cancer. The expression of ASC is downregulated in metastatic melanoma as well (210). Despite overexpression of ASC did not quite influence the progression of melanoma, silence of ASC by short hairpin RNA demonstrated tumor suppressing effects in metastatic melanoma. However, silence of ASC in different phases exerts different effects (210). When ASC was silenced in primary melanoma, it led to reduced cell death and increased cell viability. Melanomagenesis is likely to occur due to enhanced phosphorylation of IκB kinase and the activation of NF-κB pathway. On the contrary, silence of ASC in metastatic melanoma led to suppression of NF-κB pathway as well as carcinogenesis. Therefore, correlated to the secreting features of IL-1β, ASC also exhibits a dual role in different phases (210). In primary melanoma, the secretion of IL-1β needs abundant external stimuli. ASC relatively inhibits NF-κB pathway and melanomagenesis. However, in metastatic melanoma, the secretion of IL-1β is in autonomous manner, ASC promotes melanomagenesis through enhanced activation of NF-κB pathway. It is speculated that the decreased level of ASC in metastatic melanoma leads to extrinsic competition over limited ASC among different pathways, which results in enhanced inflammasome-dependent secretion of IL-1β and autoactivation of NF-κB pathway.

Genetic analysis also reveals the correlation between inflammasomes and melanoma. In a Swedish case–control study, NLRP3-rs35829419 and NLRP-rs12150220 are associated with nodular melanoma (211). NLRP3-rs35839419 is identified as a gain-of-function SNP because the level of IL-1β in this SNP tends to be elevated (211). Melanoma patients with such SNP showed resistance against T lymphocytes.

Inflammasome components can also function independently of inflammasomes. For examples, TAMs, IFN-γ-producing CD4^+^ and CD8^+^ T lymphocytes are responsible for NLRC4-induced cancer suppression, which provides a new direction in inflammasome-component-related studies (212).

### Other Cancers

Oncogenic viruses are important biological insults linking inflammation to cancer. *Kaposi’s sarcoma-associated herpes virus* and *Epstein–Barr virus* (EBV) can activate IFI16 inflammasome, a member of ALR family, which is able to detect viral DNA in the cytoplasm as well as in the nucleus (213). EBV is associated with the incidence of Burkitt’s lymphoma, Hodgkin’s lymphoma, and nasopharyngeal carcinoma (213). The expression of NLRP3, AIM2, and RIG-I inflammasomes was increased in EBV-associated cancerous tissues (214). EBV genomic DNA and EBV-encoded small RNAs are able to activate AIM2 and RIG-I and induced the secretion of IL-1β (214). The anticancer effects depend on the immunostimulatory neutrophils (214). The infiltration of TAMs improves prognosis in patients with EBV-induced nasopharyngeal carcinoma (214). To overcome the immune responses, EBV miRNA can bind to 3′-untranslated region in NLRP3, which is also the binding site of miRNA-223. By this way, EBV miRNA can induce a miRNA-223-like effect to inhibit the accumulation of NLRP3 and the production of IL-1β (215). Moreover, EBV miRNA can be transported through exosomes to dampen NLRP3 inflammasome functions in the surrounding healthy cells (215).

Another typical example of oncogenic viruses is HPV. Similarly, inflammasome components like IFI16, AIM2, IL-1β, and caspase-1 are upregulated in HPV-infected tissues (216, 217). And interestingly HPV16 E6 protein is able to decompose proIL-18 in proteasome-dependent manner, which is recognized as an evolutionary escaping strategy of HPV (218). The activation of inflammasome benefits the clearance of viruses and the regression of carcinogenesis.

The role of NLRP3 inflammasome in hepatitis virus infection has also been characterized. The proliferation of HCV in host cells results in the production of ROS, which subsequently activates NLRP3 inflammasome (219). Increased level of IL-18 in peripheral blood provided protection against expansion of infection, which is applied to both HCV and HBV infection (220). The expression of AIM in peripheral blood mononuclear cells was enhanced in acute hepatitis B compared with chronic hepatitis B (221). It suggests impaired clearance of viruses and suppressed immune responses is associated with chronic infection of hepatitis B. The expression of NLRP3 inflammasome components was also significantly decreased in hepatocarcinoma, implicating a negative correlation with hepatocarcinoma progression (222).

*Human T-cell leukemia virus type 1* (HTLV-1) retrovirus is associated with severe adult T-cell leukemia (223). Recently, genetic analysis revealed the association between NLRP3 inflammasome and HTLV-1 infection. Based on data from northeastern Brazilian population, scientists confirmed NLRP3-rs10754558 G/G is less susceptible to HTLV-1 infection (224). Other polymorphism in NLRP1 and NLRP3 were also reported in HTLV-1 infected patients (224).

**Figure 4:**
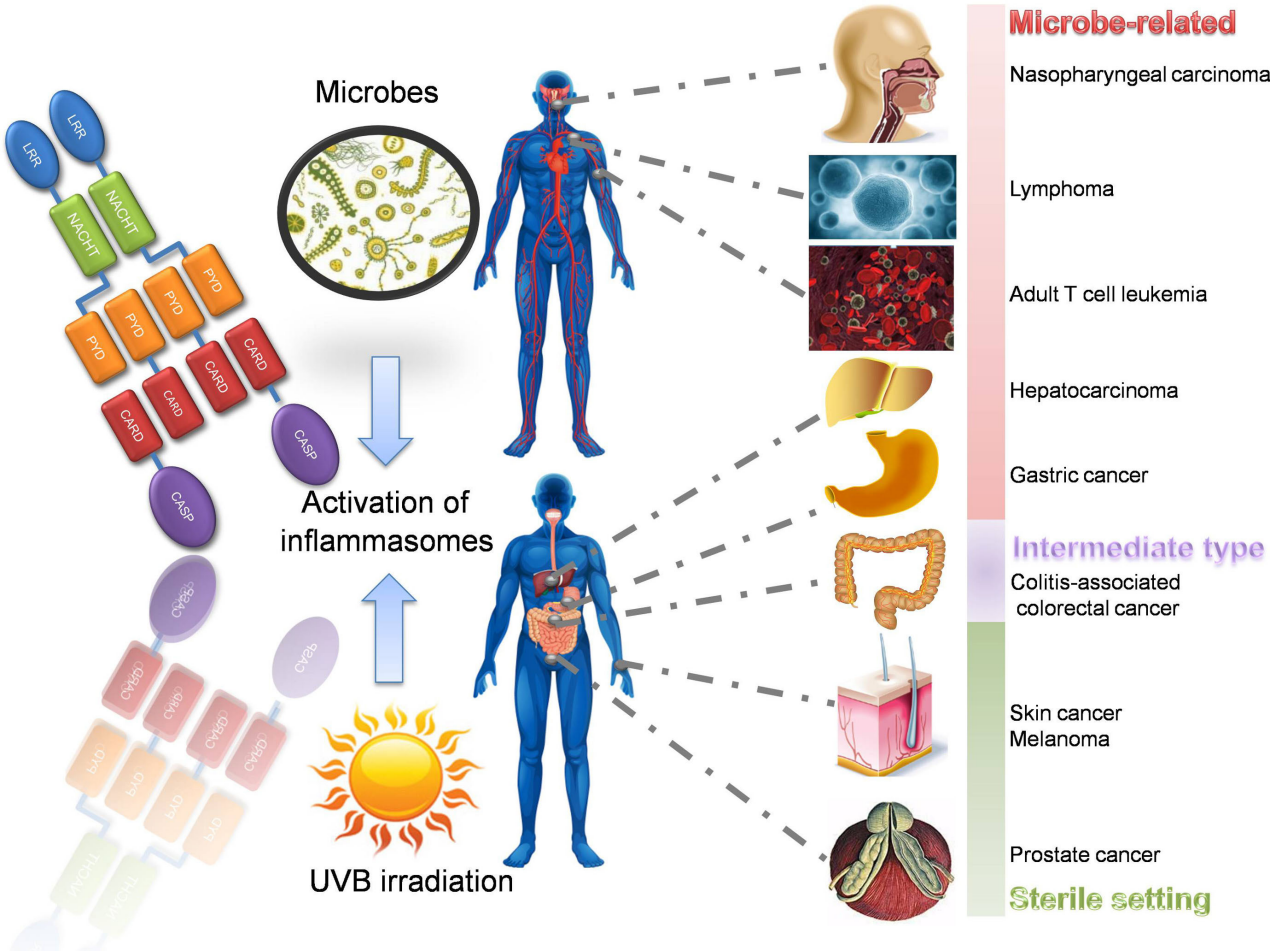
**Inflammasome-related inflammation-induced cancers**. There are various inflammation-induced cancers associated with inflammasomes. According to their pathogenesis and precipitating factors, they can be divided into microbe related, intermediate type, and sterile inflammation related. Take male as an example, microbe-related cancers include nasopharyngeal carcinoma (EBV), Burkitt’s lymphoma (EBV), adult T cell leukemia (HTLV-1), hepatocarcinoma (HCV), and gastric cancer (*Helicobacter pylori*). Colitis-associated colorectal cancer is a special type between microbe-related and sterile type, since colonic microbes and abnormal immune responses both contribute to its pathogenesis. Sterile inflammation-induced cancers include skin cancer due to the overdose UBV irradiation, melanoma, and prostate cancer. LRR, leucine-rich repeat; NACHT, nucleotide-binding and oligomerization domain; PYD, pyrin; CARD, caspase recruitment domain; CASP, caspase-1; EBV, *Epstein–Barr virus*; HTLV-1, *human T-cell leukemia virus type 1*; HCV, *hepatitis C virus*; UVB, ultraviolet radiation B.

Men, especially senile men are at high risk of prostatitis and prostate cancer. A 5-year follow-up study pointed out nearly 20% of prostate cancer develops from chronic inflammation (225). Studies show serum IL-18 is much higher in prostate cancer biopsies compared with healthy controls and benign prostatic hyperplasia (226). Also, the expression of AIM2 is significantly reduced, although IFN treatment can partly improve it (227). Similar to AIM2, ASC is constitutively downregulated in prostate cancer specimens due to hypermethylation of the gene promoter (228). The role of caspase-1 in prostate cancer is contradictive. Caspase-1 is also downregulated in prostate cancer which is speculated to prolong the cancerous cell life span (229). On the other hand, since anti-androgen treatment led to anticancer effects in early stage prostate cancer and suppression of caspase-1 at the same time, it is possible that caspase-1 plays a pro-cancer role in the secretion of proinflammatory cytokines to facilitate cancer aggressive invasion (230, 231). In a word, dominance of the dual effects of caspase-1 may depend on the stage of cancers and specific cellular compartments in which it functions. Therefore, it is not hard to conclude that inflammasomes play important and complicated roles in inflammation-induced cancers (Figure 4).

## Conclusion and Perspectives

Inflammasomes provide us with a brand new platform to explore the secrets of inflammation. Diverse types of inflammasomes reflect strong adaptability and flexibility of the human body to respond to complicated life activities. The roles of inflammasomes in inflammation-induced cancers are intricate. In one way, it can promote carcinogenesis through the extrinsic pathway and facilitate the progression and metastasis of cancer by vicious cancer microenvironment. On the other hand, proper inflammation and pyroptosis mediated by inflammasomes are necessary for proper control of tumor development. Meanwhile, inflammasomes and their components are important regulators for internal homeostasis, protecting healthy tissues against cancers. Inflammasome is a double-edged sword in cancers. Inflammasomes and their components may exhibit very distinct effects in different diseases, even different stages of exactly one disease. When it refers to a specific disease, experimental results should be interpreted individually, especially when clinical outcomes are concerned. The phase-dependent effects of inflammasomes and their components need to be paid more attention to in the future studies.

Inflammasome components have the potential to be biomarkers in malignancies, demonstrating the dynamic development of carcinogenesis as well as metastasis. The ideal biomarkers are supposed to have profound correlation with cancers and easy to be detected during early stage. Accordingly, the adaptor protein ASC and certain inflammasome proteins like NLRP3 are considerable candidates (232, 233). Several studies implicated the alteration of IL-1β along the development of various cancers and proposed IL-1β in saliva as a predicting biomarker for cancer progression (234). It was shown IL-1β is easier to be detected in saliva compared with serum. The use of saliva biomarker has been practiced in many cancers like breast cancer, pancreatic cancer, and salivary gland cancer (234). Anyway, the operability and specificity of inflammasome biomarkers need more considerations and assessments before they are applied to clinical practice.

Inflammasome-related therapy has become an emerging test field for cancer treatments recently. Several molecules targeting caspase-1 or IL-1β pathway have been developed. Some of them have already got access to clinical trials. Anakinra, a gene-recombinant antagonist of IL-1R, improves the prognosis of patients with melanoma (235). In bacteria-mediated cancer therapy, attenuated *Salmonella* activates anticancer defense of the host through NLRP3 inflammasome activation, which is triggered by the damage signals and intercellular interaction with macrophages (236). Inhibitors of inflammasomes and their products are also used to attenuate therapeutic side effects of chemotherapies. IL-1β inhibitors can alleviate bleomycin-induced lung injury and the cardiotoxicity caused by anthracycline (237, 238). Blocking of IL-1 pathway is also useful in pain management of osteolytic cancer metastasis (239, 240). Inflammasome and its products pave a new way in the era of immunotherapy for cancers, although more researches are required to better clarify the mechanisms of immunotherapy and to adjust regiments.

## Author Contributions

CL and JZ conceptualized the scope of this review. CL wrote the review. JZ revised it.

## Conflict of Interest Statement

The authors declare that the research was conducted in the absence of any commercial or financial relationships that could be construed as a potential conflict of interest.

## Abbreviations

AIM2, absent in melanoma 2; AOM, azoxymethane; ASC, apoptosis-associated speck-like protein containing a CARD; BIR, baculovirus IAP repeat; CAC, colitis-associated colorectal cancer; CARD, caspase recruitment domain; CD, Crohn’s disease; DAMP, damage-associated molecular pattern; DC, dendritic cell; DSS, dextran sulfate sodium; HIN-200, hematopoietic IFN-induced nuclear protein with 200 amino acids; IL-1β, interleukin-1β; IL-18, interleukin-18; IL-1R, interleukin-1 receptor; IBD, inflammatory bowel disease; LPS, lipopolysaccharide; LRR, leucine-rich repeat; MDSC, myeloid-derived suppressor cell; MSU, hyaluronan and monosodium urate; NACHT, nucleotide-binding and oligomerization domain; NK cell, natural killer cell; NLR, NOD-like receptor; PAMP, pathogen-associated molecular pattern; PARP, poly ADP ribose polymerase; PRR, pattern-recognition receptor; PYD, pyrin; RNS, reactive nitrogen species; ROS, reactive oxygen species; TAM, tumor-associated macrophage; TLR, toll-like receptor; UC, ulcerative colitis; UVB, ultraviolet radiation B.
